# New structural insights into Golgi Reassembly and Stacking Protein (GRASP) in solution

**DOI:** 10.1038/srep29976

**Published:** 2016-07-20

**Authors:** Luís F. S. Mendes, Assuero F. Garcia, Patricia S. Kumagai, Fabio R. de Morais, Fernando A. Melo, Livia Kmetzsch, Marilene H. Vainstein, Marcio L. Rodrigues, Antonio J. Costa-Filho

**Affiliations:** 1 Laboratório de Biofísica Molecular, Departamento de Física, Faculdade de Filosofia Ciências e Letras de Ribeirão Preto, Universidade de São Paulo, Ribeirão Preto, SP, Brazil; 2 Departamento de Física e Informática, Instituto de Física de São Carlos, Universidade de São Paulo, São Carlos, SP, Brazil; 3 Departamento de Física, Centro Multiusuário de Inovação Biomolecular, Instituto de Biociências, Letras e Ciências Exatas, Universidade Estadual Paulista Júlio Mesquita, São José do Rio Preto, Brazil; 4 Centro de Biotecnologia, Federal University of Rio Grande do Sul, Porto Alegre, Brazil; 5 Fundação Oswaldo Cruz - Fiocruz, Centro de Desenvolvimento Tecnológico em Saúde (CDTS), Rio de Janeiro, Brazil; 6 Instituto de Microbiologia Paulo de Góes, Universidade Federal do Rio de Janeiro, Rio de Janeiro, Brazil

## Abstract

Among all proteins localized in the Golgi apparatus, a two-PDZ (PSD95/DlgA/Zo-1) domain protein plays an important role in the assembly of the cisternae. This Golgi Reassembly and Stacking Protein (GRASP) has puzzled researchers due to its large array of functions and relevance in Golgi functionality. We report here a biochemical and biophysical study of the GRASP55/65 homologue in *Cryptococcus neoformans* (CnGRASP). Bioinformatic analysis, static fluorescence and circular dichroism spectroscopies, calorimetry, small angle X-ray scattering, solution nuclear magnetic resonance, size exclusion chromatography and proteolysis assays were used to unravel structural features of the full-length CnGRASP. We detected the coexistence of regular secondary structures and large amounts of disordered regions. The overall structure is less compact than a regular globular protein and the high structural flexibility makes its hydrophobic core more accessible to solvent. Our results indicate an unusual behavior of CnGRASP in solution, closely resembling a class of intrinsically disordered proteins called molten globule proteins. To the best of our knowledge, this is the first structural characterization of a full-length GRASP and observation of a molten globule-like behavior in the GRASP family. The possible implications of this and how it could explain the multiple facets of this intriguing class of proteins are discussed.

The Golgi apparatus is a highly dynamic organelle responsible for sorting out proteins and other biomolecules to the cell surface and to the extracellular milieu^1^. The Golgi assembles in its characteristic pile structure, which is important for the correct post-translational modifications of proteins^1^,^2^. The structural organization of the cisternae into stacks and their lateral connection, building the Golgi ribbon, require a family of proteins called Golgi ReAssembly and Stacking Proteins (GRASP). GRASP is a caspase-3 substrate, whose cleavage contributes to Golgi fragmentation during apoptosis^3^. Two homologues in vertebrates have been previously described (GRASP55 and GRASP 65)^4^,^5^ and their functions have been associated to Golgi phosphorylation-regulated assembly/disassembly^6^,^7^, protein secretion^8^, Golgi remodeling in migrating cells^9^, among others^10^. There is only one gene for GRASP in lower eukaryotes^10^. Although the gene has been apparently lost in the Plantae, plants conserve the regular Golgi shape structured in stacks^10^, which implies that GRASPs are likely involved in other vital functions in the cell.

GRASPs can be dramatically regulated not only in a limited physical space, such as in between the cisternae of the Golgi complex, but also in the cytosol. This family of proteins must be able to correctly interact with itself in a *trans* orientation (thus preventing the *cis* oligomerization)^11^ to be accessible to the phosphorylation/dephosphorylation machinery in the cell cycle^12^ and to proteases during apoptotic times^3^. Furthermore, GRASP is involved in the direct interaction with a large number of partners/ligands in the conventional and unconventional secretion pathways^13^,^14^,^15^. Among those, the unconventional secretion of a major capsular component and the most important virulence factor^16^ of the basidiomycetes fungus *Cryptococcus neoformans*, the polysaccharide glucuronoxylomannan (GXM), has proven to be GRASP-dependent^17^. The yeast-like pathogen *C. neoformans* is the principal causative agent of cryptococcal meningitis, an opportunistic disease that kills about half million people every year worldwide^18^,^19^. Fungal infections are a threat to human health. About 1.2 billion people worldwide are estimated to suffer from a fungal disease^20^. Of these, over 1.5 million people are estimated to die^21^. The need for new antifungals is thus clear, which stimulates studies on the structural characterization of molecular targets, such as GRASP, regulating fungal virulence and/or physiology.

Structural information about GRASP is still incipient. The crystal structures of the N-terminal domain from human GRASP55^22^, *Rattus norvegicus* GRASP55^23^ and *Rattus norvegicus* GRASP65^23^ have been only recently reported. Two-PDZ domains, which form the so-called GRASP domain, and a non-conserved serine and proline rich domain, usually larger than the GRASP domain, compose the overall structure^10^. These structural data together with several *in vivo* studies aiming at functional elucidation^10^ show that GRASPs are involved in a large set of functions that likely includes a great number of interacting partners. However, the behavior of a full-length GRASP is still obscure, since its biophysical properties in solution are still unknown. As a first step towards unraveling GRASP behavior, we report here a biochemical and biophysical study of *C. neoformans* GRASP (CnGRASP) in solution. Bioinformatic analysis, static fluorescence and circular dichroism spectroscopies, calorimetry, small angle X-ray scattering, solution nuclear magnetic resonance, size exclusion chromatography and proteolysis assays were used to probe structural features of the full-length CnGRASP. Our results indicate an unexpected behavior in solution and provide information that can affect the way one thinks GRASP accomplishes its plethora of functions.

## Results

### Primary sequence analyses of CnGRASP suggest multiple disordered sites

GRASPs have a well-conserved domain at their N-terminus comprising two tandem PDZ domains (PDZ-1 and PDZ-2), a classical protein-peptide interaction domain, which is responsible for GRASP homo-oligomerization and for the attachment to the Golgi membrane^24^. The C-terminus is a Serine and Proline Rich domain (SPR domain) with a large number of phosphorylation sites in mammalian cells. The SPR domain is apparently required for GRASP regulation^24^.

The CnGRASP primary sequence [17, Broad Institute Accession No. CNAG_03291] has 256 amino acids and a theoretical molecular mass of 27551.7 Da with a composition of almost 30% of bulky, or order promoting, amino acids (I, C, L, V, W, Y and F). As a matter of comparison with ordinary soluble proteins, for Conalbumin (GenBank: CAA68468.1) this value reaches 31%, Ovalbumin (GenBank: AAB59956.1) is 33% and Bovine Serum Albumin (GenBank: CAA76847.1), 34%. Those amino acids are responsible for driving the formation of the hydrophobic core in globular proteins.

**Figure 1:**
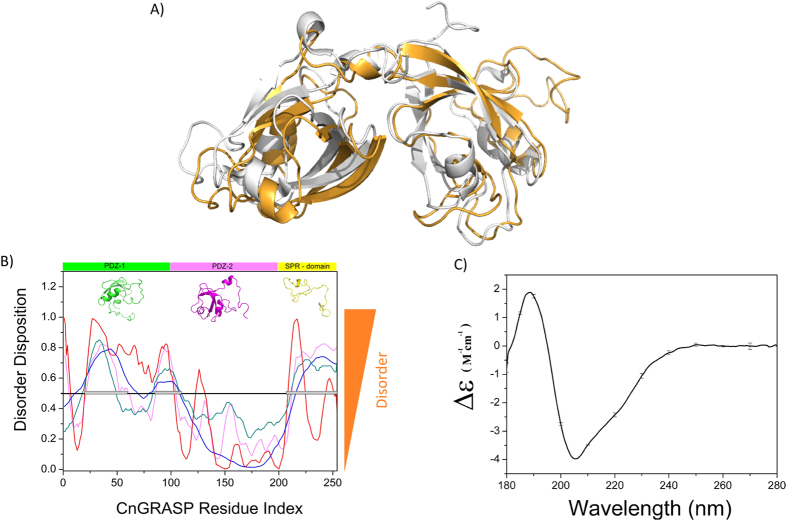
Secondary structure analyses. (**A**) CnGRASP (Orange) and GRASP55 (Gray, PDB ID 3RLE) GRASP domain alignment using Pymol. (**B**) Disorder prediction using VSL2B (Magenta), VLXT (Red), VL3 (Blue) and Ronn (Green). The black line indicates the limit above which the residue has more than 50% propensity of being disorder. Represented in the upper part of the graph are the refined predicted models of CnGRASP. The green part represents PDZ-1, the magenta represents PDZ-2 and the SPR domain is colored in yellow. (**C**) SRCD spectrum of CnGRASP in solution.

The CnGRASP GRASP domain seems to have an overall structure similar to GRASP55 (Fig. 1A), even though a much more pronounced quantity of disorder is apparently present. Despite this overall structure resemblance, the analysis of the sequences of each domain showed that PDZ-1, PDZ-2, and SPR presented variable amounts of disorder-promoting amino acids (A, G, R, D, H, Q, T, K, N, M, S, E and P). PDZ-2 has the highest percentage of order promoting amino acids (40%), whereas SPR has only 23% in total length, being mainly composed of prolines, arginines and serines, a general feature within the GRASP family. It is worth noting that the amount of proline, a well-known structure breaker, is almost 8% of the total protein amino acid content and that is much larger than values found in structured proteins such as human serum albumin (4.1%), human lysozyme (1.5%) and *Bos taurus* Ribonuclease A (3.2%). The CnGRASP primary sequence was also analyzed using the Uversky plot^25^. The mean net charge and mean hydrophobicity values place CnGRASP in the native side of this charge-hydrophobicity phase space (Figure S1A) similar to well-structured proteins, and the same behavior observed for other GRASPs (Figure S1B). These analyses indicated that CnGRASP has the necessary amino acid content to be a native and well-structured protein, although presenting non-homogeneous distribution of the bulky disorder-promoting amino acids.

The prediction of local disorder can be performed with reasonable accuracy^26^. Analyzing factors such as local amino acid composition and hydropathy, protein disorder predictors classify each residue within a sequence with a propensity to be either ordered or disordered. Through the use of a combination of such programs, including VSL2B, VLXT, VL3 and Ronn, disordered regions in CnGRASP were assessed. This analysis clearly demonstrated the high propensity of the SPR domain to be intrinsically disordered (Fig. 1B). The behavior of the first PDZ domain is of particular interest since it is responsible for the *trans*-oligomerization in vertebrates and possibly in *C. neoformans.* PDZ-1 contains regions having a high propensity for local disorder (Fig. 1B), a behavior that is not observed for CnGRASP PDZ-2, which remains structured over most of its sequence. The reason why PDZ-1 follows the disorder propensity observed above, and PDZ-2 does not, is still an open issue. Several reports suggest the concept of cargo-specificity in the GRASP-mediated tethering of cargo molecules^10^,^15^. The structural flexibility observed in intrinsically disordered proteins can confer high specificity with low affinity for molecular recognition, allowing it to interact with multiple partners^27^, a feature that could be properly explored by the PDZ-1 in order to interact with the cargo proteins during functional processes.

### Secondary structure analyses of CnGRASP revealed well-structured regions

Computational methods, as those used in the previous section, are very useful tools for protein structural analyses and, for CnGRASP, it could be predicted that multiple sites of disorder coexist with more structured regions. These observations were further investigated by including experimental methods that provided information on the structural organization of CnGRASP. Circular dichroism is a powerful technique for structural studies of proteins in solution^28^,^29^. The CnGRASP Synchrotron CD spectrum in the far-UV region (Fig. 1C) indicated that the protein is of the α/β type with the typical features of α-helical components (bands centered around 208 and 222 nm), which always show up more clearly in the CD spectra of proteins. The presence of β-strand contributions is suggested by the loss of resolution in those α-helical bands that likely come from the admixture of the β–associated band around 215 nm, which was not resolved in the spectrum. To quantify the secondary structure content, deconvolution algorithms can be used^30^,^31^. For CnGRASP, DICHROWEB^32^ was employed and the results of the deconvolution are presented in Table S1. DICHROWEB utilizes three distinct algorithms to evaluate the contents of secondary elements, thus providing their mean values. On the basis of this analysis, the secondary structure content appeared to be mostly due to non-helical elements such as β-sheets (~22,3%) and to structures in the form of loops (~18%) and disordered regions (~39). It is important to notice that the predicted amount of disorder reached high values, which are consistent with the bioinformatic results and with the secondary structure prediction (Table S2).

### Quaternary structure analyses of CnGRASP

Size exclusion chromatography (SEC) is a hydrodynamic technique widely used to study protein oligomerization states. A calibration curve can be prepared with known globular proteins and if the unknown protein is also globular, the partition coefficient (k) can be directly related to its molecular mass. The elution pattern of CnGRASP (Figure S2A) resulted in a k value of 0.422. From this, we can infer an apparent molecular mass in solution of 78 kDa, indicating that the protein has a quaternary structure that seems to be a trimer in solution (Figure S2B). These results contradict those obtained for the homologous of CnGRASP in mammals and are also in disagreement with the proposed mechanism of membrane tethering in which a GRASP dimer is fundamental^10^,^23^. Barr *et al*. described that the apparent molecular mass of GRASP65 measured by gel filtration on a Superose 6 HR10/30 column (Pharmacia) indicated a large discrepancy with respect to the expected mass of a dimer^33^, even though GRASP65 has proved to be a dimer in solution^24^. Furthermore, Wang^34^ and Barr^4^ found in coexpression experiments that GRASP65 behaved as a dimer in solution. An aberrant hydrodynamic behavior is usually a very solid indication of protein disorder^35^. The presence of intrinsically disordered regions increases the average volume of occupation, which would make these proteins slightly “larger” than expected.

**Figure 2:**
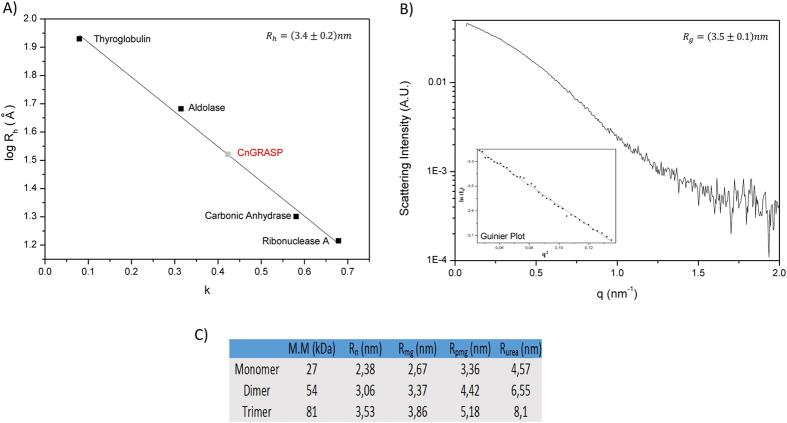
Hydrodynamic properties of CnGRASP. (**A**) Linear dependence of R_h_ as function of the partition coefficient obtained from SEC. Standard proteins were obtained from Gel Filtration Calibration Kit LMW/HMW (GE Healthcare). (**B**) SAXS data obtained for CnGRASP and the same data in the region of Guinier approximation (inset). The range used was between 0.759 and 1.294 qR_G_, values obtained using PRIMUS in ATSAS package. (**C**) Theoretical values of molecular mass (MW) and the theoretical associated hydrodynamic radius expected for a native globular protein (R_n_), molten globule (R_mg_), pre-molten globule (R_pmg_) and denatured protein in urea (R_urea_). The calculated hydrodynamic radii were based on the theoretical molecular mass of each oligomer using the experimental equations in ref. 37.

SEC can also be used to recover the hydrodynamic radius (R_h_) of an unknown protein in solution^36^ and, in the case of CnGRASP, resulted in the R_h_ value of (3.4 ± 0.2) nm (Fig. 2A). The expected R_h_ value for a dimer of CnGRASP, if it were a globular protein, would be close to 3 nm (Fig. 2C) as calculated according to ref. 37. Instead, the R_h_ value calculated in Fig. 2A agrees with the expected value for a dimer of a molten globule protein (Fig. 2C).

Another method also used to gain information on the quaternary structure of proteins is the Siegel and Monte model^38^, which has been applied in oligomerization studies of a large number of proteins in solution^36^,^37^,^38^,^39^,^40^,^41^, including IDPs^42^,^43^. The method relies on two hydrodynamic parameters (hydrodynamic radius and sedimentation coefficient) that are combined to obtain the molecular mass of the protein in solution without the need for the globularity premise. The final equation is: *M* = s*N*_*0*_(6πη*R*_*h*_)/(1 − *ν*_2_*ρ*) , where M is the molecular mass, s is the sedimentation coefficient, *ρ* is the solvent density, *η* is the viscosity of the solvent, *N*_0_ is the Avogadro’s number, *R*_*h*_ is the hydrodynamic radius and ν_2_ is the partial specific volume of the protein. For the calculation, *η* was set to 0.001 *Pa.s* (water density) and ν_2_*ρ* equals to 0.73 (using the standard protein *ν*_2_ = 0.73 cm^3^/*g* and (*ρ* = 1 *g*/*cm*^3^). The hydrodynamic radius of CnGRASP is (3.4 ± 0.2) nm (as found from SEC data) and the sedimentation coefficient was estimated using zone sedimentation in a sucrose gradient by comparison to standard proteins of known s (carbonic anhydrase, bovine serum albumin and conalbumin) (Figure S3). The value obtained for s was close to 4.2 S, which implies an estimated molecular mass of 60 kDa, close to the theoretical value expected for a dimer (54 kDa).

We also evaluated the R_h_ value using DLS experiments (inset in Figure S2A). In this case, the result showed a signal centered at 3.9 nm that accounts for 99% of the measured volume. This signal has a relative polydispersity of 18%, which indicates that this peak is monodisperse and that there is one major protein population. The apparent discrepancy between the R_h_ values determined from SEC and DLS experiments is probably due to the use, in the DLS calculation, of a poorly defined viscosity value. The buffer used during CnGRASP preparation contains 10% V/V glycerol, thus increasing the solution’s viscosity. This makes the molecules diffuse slower and that is accounted for during the analysis as a “larger” molecule than it truly is. From this R_h_ value, we obtained a molecular mass of 68.9 kDa, which is more consistent with a dimer than a trimer, since the mass values determined in this sort of calculation are usually deviated to greater values. This result confirmed that the globularity deviation observed for CnGRASP is not caused by an unexpected quaternary structure as initially suggested by the estimated mass determined from our SEC experiment.

Another related measure of the size of a molecule is its radius of gyration (R_g_). For CnGRASP, the value of R_g_, determined using Synchrotron Small Angle X-Ray Scattering (in the Guinier approximation) was (3.5 ± 0.1) nm (Fig. 2B). This R_g_ value is consistent with the one obtained from the distance distribution function calculated from the experimental X-ray scattering data (3.7 nm, using GNOM, from ATSAS package) in the range of concentrations used. Furthermore, Narang *et al*.^44^ used a computational based method and structures available in the protein data bank (PDB) to construct a linear function relating the values of R_g_ with the total number of amino acids in a protein sequence (http://www.scfbio-iitd.res.in/software/proteomics/rg.jsp). As the PDB database is mostly comprised of structured and globular proteins, the value obtained experimentally by SAXS for CnGRASP (3.5 nm) does not follow the R_g_ of 2.4 nm obtained using the Jayaram model, again suggesting that CnGRASP does not behave as a completely globular protein.

Although both R_g_ and R_h_ can be used as parameters to assess the degree of compactness of proteins, they refer to different aspects of the molecular structure. The value of R_g_ is related to the geometrical dimensions of the molecule, whereas R_h_ is related to the hydrodynamic properties. A constant known as the *Q*-factor is an indicative of molecular conformation^45^,^46^. The *Q*-factor is given by 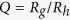, being equal to 0.775 for a compact sphere or close to 0.8 for globular proteins, and 1.51 for a theoretical random coil^45^. For proteins with pre-molten globule conformation, the values are close to 1, and for molten globule they are between 0.7 and 0.93, both being closer to the values observed for compact proteins^45^. The values determined above for CnGRASP yielded a *Q*-factor equal to (1.03 ± 0.09), which is closer to what is expected for a protein either in a molten globule or in a pre-molten globule conformation.

**Figure 3:**
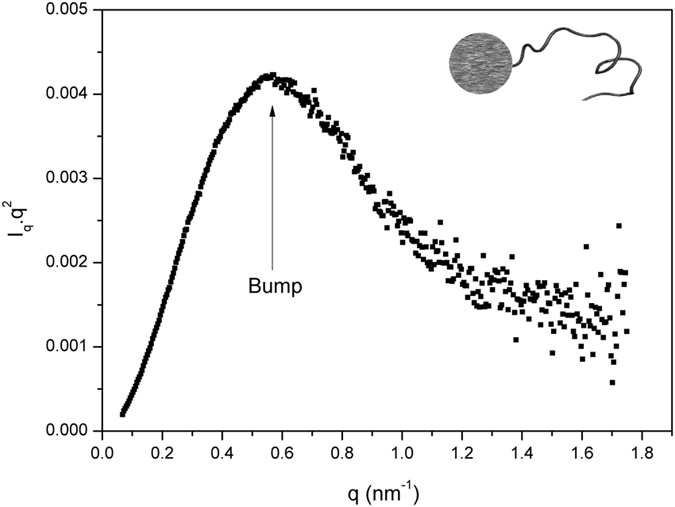
Solution behavior of CnGRASP. SAXS data viewed in a Kratky plot for CnGRASP. The bump observed for CnGRASP at q value close to 0.6 nm^−1^ is indicative of a compact structure. The model of a compact structure plus a coil one (located in the inset) was based on ref. 50.

The information needed to distinguish between molten globule and pre-molten globule can be extracted from the SAXS data seen in the Kratky plot^26^, whose shape is sensitive to the conformational state of the scattering particles and to their degree of flexibility^26^,^47^,^50^. The scattering curve in the Kratky plot has a characteristic maximum when the protein is compact, such as in the globular or in the molten globule states, while this maximum is absent in the pre-molten globule and unfolded states^48^,^49^. A very useful pattern recognition scheme is presented by Rambo and Tanier^50^ and following such scheme we determined that the shape of the SAXS data in the Kratky plot for CnGRASP (Fig. 3) is compatible with the data from a partially folded protein containing a compact and a disordered domain, which is exactly the expected structural arrangement for CnGRASP (the GRASP domain linked to the SPR domain)^50^. We can then conclude that rather than behaving as a pre-molten globule protein, CnGRASP has a molten globule-like behavior.

**Figure 4:**
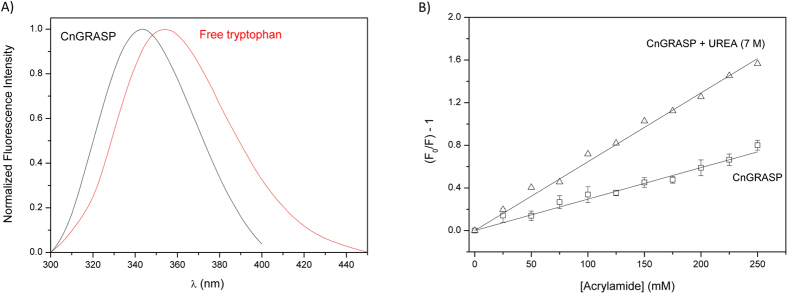
Hydrophobic core accessibility using tryptophan fluorescence. (**A**) Fluorescence emission of CnGRASP with an excitation radiation of 295 nm. (**B**) Stern-Volmer plot of CnGRASP and a denatured state condition (with 7 M urea) using acrylamide as a collisional quencher.

Fluorescence experiments using the tryptophan residues in CnGRASP were also used to strength the conclusions drawn from the SAXS data in the Kratky plot. Tryptophan amino acids can be selectively excited using a monochromatic radiation of 295 nm. CnGRASP has four native tryptophan residues that should all be in similar structural environments if CnGRASP is indeed a molten-globule protein, as suggested by the results presented above. Similar environments in a highly flexible structure will lead to high tryptophan accessibility to the solvent^35^. For solutions of free tryptophan in water, the wavelength of maximum emission in the fluorescence spectrum occurs around 350 nm (354 nm in Fig. 4A)^51^. Tryptophans located in the hydrophobic cluster of proteins show a blue shift in that wavelength to values around 320 nm^51^. CnGRASP has an emission maximum close to 344 nm (Fig. 4A), indicating that the tryptophans are neither fully exposed to the solvent nor in a very hydrophobic environment, therefore implying a certain degree of structuration of CnGRASP. Moreover, all tryptophans apparently have the same high accessibility to the solvent based on the linear Sterm-Volmer plot obtained from a quenching experiment using acrylamide (Fig. 4B). Tryptophan residues in a hydrophobic environment (fluorescing at 344 nm) and still with high and similar solvent accessibility (Stern-Volmer plot) is consistent with molten globules, mainly because of their less compact and still structured conformations and high structural flexibility^52^.

### Low cooperativity of unfolding

**Figure 5:**
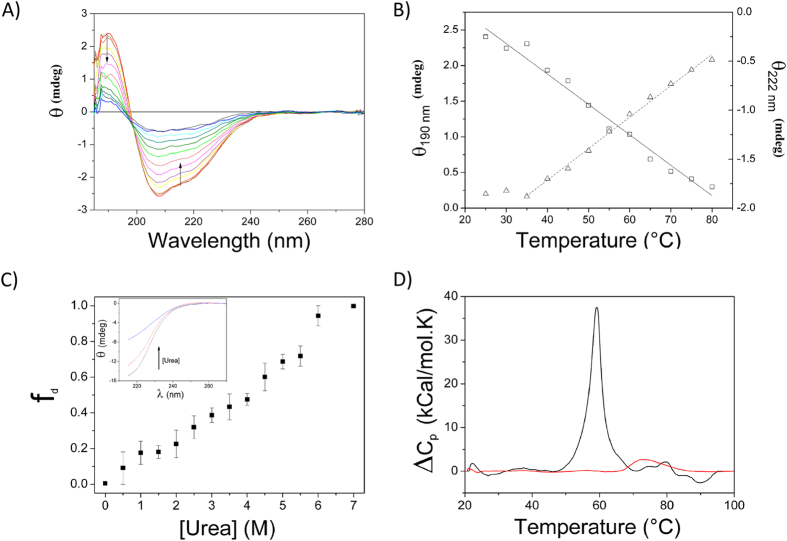
CnGRASP denaturation profile under thermal and chemical perturbation. **(A**) Thermal induced unfolding monitored using SRCD. The arrows indicate the direction of spectral changes upon temperature increases. (**B**) Plots of the peak magnitudes at 190 (square) and 222 nm (triangle) during the heating process. (**C**) Urea-induced unfolding of CnGRASP monitored by far-UV. CD. The unfolding fraction f_d_ was calculated from the CD intensity at 220 nm (inset). (**D**) Thermal unfolding of CnGRASP (red line) and Conalbumin (black line) monitored by DSC.

The free energy landscape for protein folding (or unfolding) can dramatically change when structured proteins and IDPs are compared^53^. A plateau due to the high flexibility of the structure can represent the free energy pattern for IDPs. This leads to a monotonic decrease of protein structure content and thus to a low cooperativity in the protein fold/unfold transition induced by a denaturant agent^53^. CnGRASP followed this behavior as assessed by chemical and thermal denaturation experiments performed using CD spectroscopy (Fig. 5A–C). The CD values at 190 nm and 222 nm are typically associated with helical structures and their temperature variation showed a low cooperative thermal unfolding (Fig. 5B). Moreover, there was no detected gain in protein secondary structure induced by the temperature increase, a feature previously observed for fully extended IDPs^35^,^46^. The thermal unfolding is irreversible, as can be observed in Fig. 5B, since the SRCD spectrum measured after the system was allowed to cool down is different compared to the initial spectrum. The low cooperativity in the thermal induced unfolding was also confirmed using DSC with conalbumin as a control (Fig. 5D).

**Figure 6:**
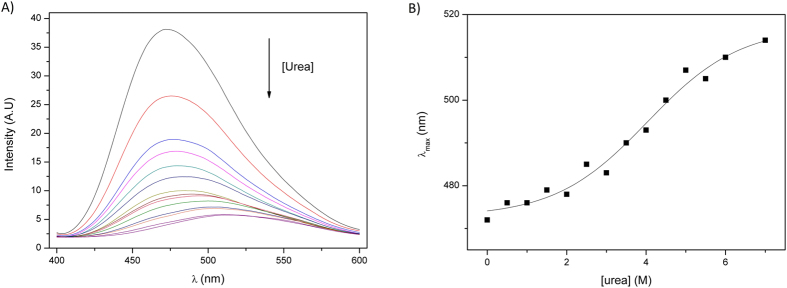
Urea induced unfolding of CnGRASP monitored using the ANS fluorescence. (**A**) The decrease in fluorescence intensity is an indicative of the ANS microenvironment change, during urea titration, from a non-polar environment (hydrophobic core) to a polar environment (water exposed). The high fluorescence intensity in the beginning is an indicative of high ANS affinity. (**B**) Variations of the wavelength of maximum emission intensity as a function of urea concentration, again showing a poorly cooperative unfolding process.

The chemical unfolding was investigated by means of urea titration experiments. It is possible to observe a monotonic decrease of the ellipticity at 220 nm (Fig. 5C), which has a linear instead of the sigmoid-like behavior usually observed for well-structured proteins, indicating a low cooperativity also during chemical unfolding as probed by CD. An equivalent experiment performed using static fluorescence and monitoring the fluorescence of 8-anilino-1-naphthalenesulfonic acid (ANS) led to the same conclusion (Fig. 6). ANS is a fluorescent probe widely used in the identification of accessible hydrophobic sites in proteins^54^,^55^. The change in the fluorescence pattern upon increasing urea concentration reflects changes in the microenvironment of ANS from a hydrophobic medium (interior of the protein - structured) to a polar environment (exposed to the solvent after denaturation) (Fig. 6A). The shift in the wavelength of maximum emission occurred in a low cooperativity way (Fig. 6B), indicating that the exposure of the hydrophobic core also followed the behavior observed for the secondary structures during chemical denaturation. High fluorescence intensity in the beginning is a result of high ANS affinity (Fig. 6A), also a property observed for proteins in the molten globule state^35^. Altogether, these results suggest that the secondary structure elements in CnGRASP are not well stabilized by tertiary contacts such that the protein loses them in a poorly cooperative way when unfolded by a denaturant agent. The high ANS affinity shows that CnGRASP structure is flexible enough to allow access to its partially hydrophobic interior, which is in agreement with the tryptophan fluorescence data.

### High structural flexibility

**Figure 7:**
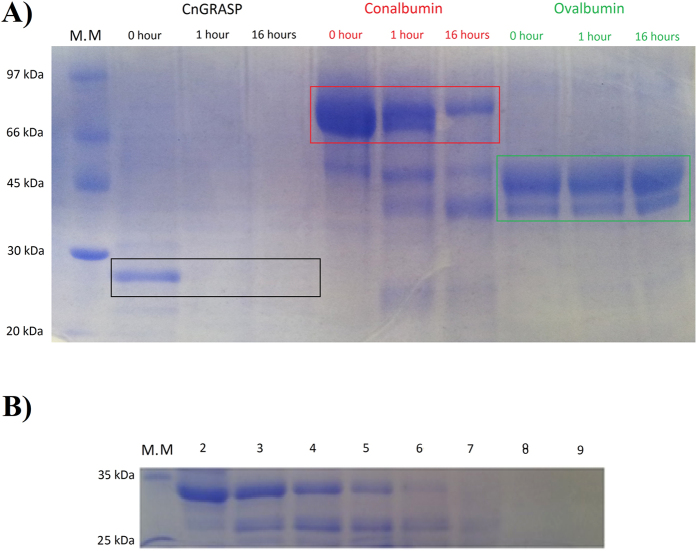
Proteolytic assay with Trypsin at room temperature. **(A**) The protein:trypsin ratio was 20:1 and the collection times were 0, 1 and 16 hours after adding trypsin to the solution. (**B**) The protein:trypsin ratio was 100:1 and the collection times were 0, 10, 15, 20, 25, 30 and 60 minutes (lanes 2–8) after adding trypsin to the solution. A protein weight standard was applied in lane 1. The reaction was stopped by adding SDS-PAGE loading buffer and heating to 95 °C for 10 minutes.

The structural flexibility of CnGRASP was further investigated by enzymatic digestion, which has been a widely used method to elucidate local disorder and flexibility in proteins. Fontana *et al*. showed that local flexibility and not only solvent exposure is the determinant factor for an efficient proteolytic activity^26^,^56^, whereas stable secondary structures usually have a protective effect against proteolytic cleavage^35^. The proteolytic assay was performed with CnGRASP and the structured proteins conalbumin and ovalbumin as controls. Trypsin is a protease that cleaves peptide chains mainly at the carboxyl side of the amino acids lysine or arginine, except when they are linked to proline^57^. All the proteins tested have comparable multiple sites predicted to be cleaved by trypsin, allowing then the identification of cleavage sites solely based on the flexibility of the proteic substrate. The results of the procedures are outlined in Fig. 7. Three incubation times with trypsin (0, 1, and 16 h) were used for each protein tested. Aliquots of each sample were then collected and visualized in SDS-PAGE (Fig. 7A). It was possible to notice the high sensitivity of CnGRASP to proteolysis since no band was seen on the gel after 1 h of incubation with trypsin. This observation validated our results concerning structure ordering and flexibility. Well-structured proteins can resist to proteolysis for long periods as observed with conalbumin and ovalbumin. Conalbumin generated a low content of protein digests with significant amounts of intact bands even after 16 h of incubation. Similarly, ovalbumin showed no signs of susceptibility to proteolysis. To assess CnGRASP hydrolysis in more detail, a 5-fold higher ratio of CnGRASP:Trypsin was tested. Even though protein degradation occurred slower than observed under the conditions described before, it was already impossible to detect the presence of intact CnGRASP by SDS-PAGE after 30 minutes (Fig. 7B).

### NMR studies prove the molten globule-like behavior of CnGRASP

**Figure 8:**
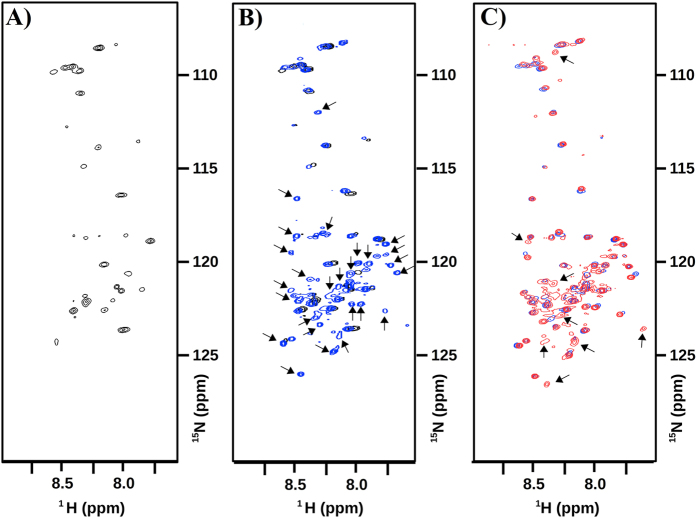
NMR data analyses of CnGRASP. **(A**) ^15^N-^1^H HSQC spectrum of ^15^N-labeled CnGRASP at 20 °C. (**B**) Superposition of ^15^N-^1^H HSQC CnGRASP spectra (black) with the same sample plus 2 M Urea (blue). (**C**) Superposition of ^15^N-^1^H HSQC CnGRASP spectra plus 2 M area (blue) with the same sample with 4 M Urea (red). The black arrows indicate the new signals that start to appear.

Molten globule structures have a very particular behavior when NMR is used to measure the signals arising from the amide groups^58^,^59^. The conformational fluctuations allowed by the lack of stable tertiary contacts create a fluctuating ensemble of structures that inter-convert in a millisecond to microsecond time scale^58^. In this time scale, the amide resonances are very broad and difficult to be detected in the ^15^N-^1^H HSQC spectrum^58^,^60^. CnGRASP ^15^N-^1^H HSQC spectra are shown in Fig. 8. It is possible to observe a very characteristic pattern of intrinsically disordered proteins: the presence of few peaks (around 50) collapsed in a very small region (close to 1 ppm) (Fig. 8A). Since the assignment of the peaks has not been performed yet for CnGRASP, we could just speculate that those few resonance peaks would come from the SPR domain and/or other regions predicted as intrinsically disordered in the protein (see above). In an indirect CD result, we measured the CD spectrum of the full-length CnGRASP and subtracted from it the CD spectrum of the isolated GRASP domain (Figure S4). The subtracted spectrum, presumably corresponding to the spectrum of the SPR domain, had the usual pattern observed for a poliproline-2 structure, characteristic of extended IDPs^28^,^35^,^42^. This result supports the conclusions drawn from our NMR data regarding the origin of the peaks observed in the ^15^N-^1^H HSQC as being from the SPR domain.

It has been previously observed that it is possible to play with the molten globule conformation equilibrium by disturbing the sample with chaotropic agents or with changes in the temperature^58^,^60^,^61^. For molten globules, when the concentration of a chaotropic agent, such as urea, is increased, the resonance lines start to appear in the spectra, but still with low chemical shift dispersity^58^. When a 2 M urea solution is added to the GRASP sample (Fig. 8B), the number of resonance lines seen in the spectrum is increased, and, at 4 M urea (Fig. 8C), peaks are still appearing similarly to what has been observed for other molten globule structures, such as α-lactoglobulin^60^ and staphylococcal nuclease^62^. These data strongly indicate that full-length CnGRASP do have a molten-globule like behavior. However, the particular condition where all the resonance peaks can be observed is still to be determined.

### Tryptophan fluorescence and local ordering

**Figure 9:**
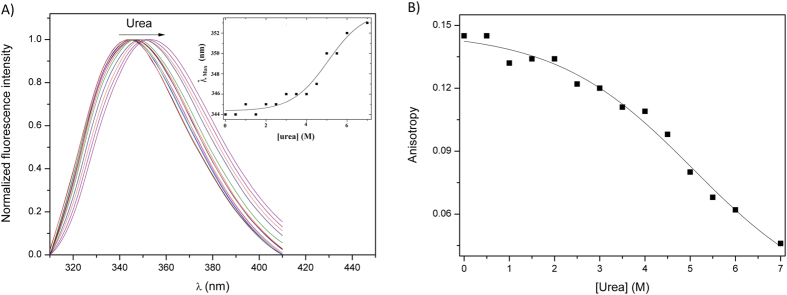
Tryptophan exposure and local secondary structure identification using urea induced unfolding. **(A**) Static fluorescence emission as a function of urea concentration. The wavelength of maximum emission intensity is plotted in the inset as a function of the urea variation. The data was fitted to a sigmoid-like function. (**B**) Static fluorescence anisotropy change during unfolding.

Three out of the four tryptophans present in CnGRASP are located in the PDZ-2 domain, which is presumably the most ordered domain of the protein. If this hypothesis is true, PDZ-2 can go through a structural transition in a more cooperative way during chemical denaturation. In fact, tryptophan fluorescence analysis showed that under increasing urea concentration, the wavelength of maximum emission red-shifted to values around 352 nm, which are characteristic of free tryptophan in solution (Figs 4 and 9). Furthermore, the decrease of the fluorescence anisotropy during urea denaturation showed that there is a relevant difference of tryptophan immobilization before and after denaturation (Fig. 9). These results suggest that, in the PDZ-2 of CnGRASP, there is a hydrophobic core, but with high solvent accessibility. The cooperativity observed in the chemical denaturation proved the existence of local ordered structures, likely around the tryptophan residues in the PDZ-2 domain. Even though the whole structure seems to be flexible, there are well-structured regions within the protein that can go through a transition in a more cooperative way. Besides that, it is possible to see that the transition is not highly cooperative and this may seem the reason why it is not resolved in other denaturation data. We should be able to observe a more cooperative transition of the whole protein if the PDZ-1 followed the same PDZ-2 behavior. However, this is not seen in our data for full-length CnGRASP and the reason why we cannot observe it is an indicative that CnGRASP has a PDZ structural asymmetry, a result that correlates well with the disordered prediction.

## Discussion

The behavior of full-length GRASPs, especially in solution, is still poorly understood. A GRASP orthologue has been described as a regulator of polysaccharide export, through non-conventional mechanisms, and related to pathogenesis in the neuropathogen *C. neoformans*^17^. Here, a series of biochemical and biophysical methods were used to assess the dynamic behavior in solution of *C. neoformans* GRASP. CnGRASP apparently has a mixture of substantial content of secondary structures with a significant amount of disordered regions as assessed by four disorder predictors and by experimental data from circular dichroism, NMR and fluorescence. In particular, the SPR domain appears as a highly disordered portion of the protein in all predictions and among all species (Figure S5), thus suggesting that the intrinsic high flexibility associated with disorder could well correlate with its role as a phosphorylation site within the protein sequence. Interestingly, PDZ-1 domain of CnGRASP also presented high disorder propensity, which could suggest an asymmetric role in functional processes, such as oligomerization, as further discussed in this section.

The hydrodynamic properties of CnGRASP pointed to an aberrant behavior of the protein in solution based on the results from SEC experiments that suggested CnGRASP would be a trimer, which was totally incompatible with the need for a dimer for correct function of the protein and in disagreement with data available in the literature. This flawed prediction from SEC was likely due to the use of globular proteins as controls and the correct behavior was only recovered when the Siegel-Monte model, which does not require the globular assumption, was used (prediction of a molecular mass of 60 kDa for CnGRASP, a value compatible with the expected dimer in solution). Therefore, the unusual migration in the reported experiments was not due to the formation of higher-order oligomers but only to the existence of parts of the protein structure that do not follow globular-like patterns.

Furthermore, SAXS and fluorescence results indicated that CnGRASP is a collapsed protein, but it lacks the typical globular behavior of many well-structured proteins. Profiles of protein denaturation do not follow the usual cooperative transition observed for well-structured proteins, as suggested by the results from different techniques (CD, SRCD, fluorescence and DSC) obtained during thermal and chemical CnGRASP unfolding. The high proteolysis sensitivity together with the SAXS Kratky plot profile showed that the protein presents high flexibility, which probably results in substantial solvent accessibility as also inferred from our quenching of fluorescence data. Overall, these results indicated that CnGRASP behaves rather as a native molten globule than as a regular globular protein in solution. The ^1^H-^15^N HSQC CnGRASP data confirmed this conclusion by showing an increasing number of resonances upon urea addition, therefore following a pattern previously reported for other molten globules. It is important to notice that the disordered/flexibility observed does not mean that CnGRASP is not functional. Recombinant CnGRASP was proved to be functional^17^ and can form dimers in solution, so flexibility does not seem to interfere in these processes. It may well be that the flexibility of the protein structure can facilitate the information propagation, making the GRASP domain more accessible to regulation.

The lack of cooperativity observed in the unfolding of CnGRASP when methods that are based on the observation of physicochemical parameters monitoring the overall protein structure suggest that the degree of disorder in CnGRASP plays a crucial role in preventing the structure as a whole to unfold as a regular globular protein. However, our bioinformatic results indicated that disorder-promoting residues are heterogeneous distributed along the protein primary sequence. Moreover, when fluorescence is used to monitor a localized region of the protein, such as the tryptophan residues’ vicinities, a more cooperative unfold is observed, which, in agreement with the theoretical predictions, indicated the presence of ordered regions in the protein structure. Interestingly, both the computational predictions and the Trp fluorescence results suggested that the more ordered portions would be located in the PDZ-2 of the PDZ domain (three of the four Trp residues in CnGRASP are located in the PDZ-2). CnGRASP structure would be formed by more disordered SPR and PDZ-1 and more ordered PDZ-2 domains. This asymmetric structural (or unstructural) behavior observed for PDZ-1 and PDZ-2 could then be responsible for the correct formation of the needed dimers during the stacking of Golgi cisternae. It has been proposed, for human GRASPs, that during the stacking process PDZ-1 interacts with the Golgi membrane through a myristoylation of a glycine residue in its N-terminal, whereas PDZ-2 binds to its receptor GM130 located on the cisternae surface. However, it has been recently shown that the GM130 C-terminal peptide can bind concomitantly with both PDZ-1 and PDZ-2, although the mode of binding involves the interaction of distinct regions of the peptide with each one of the PDZs^63^. This, again, suggests that each PDZ may have different roles in physiologically related phenomena, therefore in agreement with our conclusion on functional asymmetry of the PDZs. Moreover, during non-conventional secretion and Golgi assembly, PDZ-1 and PDZ-2 bind to different partners as previously suggested^10^,^64^. In a recent report, it has been shown that even the SPR domain can have protein partners^65^. The control of which PDZ (1 or 2) or SPR binds (or interacts) to a determined partner could be performed by means of the asymmetric order/disorder behavior of the CnGRASP domains.

The presence of IDRs in CnGRASP can also be used to understand how the protein is able to indiscriminately interact with a large number of other proteins participating in secretion pathways. GRASP can directly interact with the C-terminal of the Transforming Growth Factor-a (TGF-a) and several members of p24 family, facilitating the conventional secretion of these proteins^13^,^14^. GRASP proteins are also directly related to the unconventional secretion of a large number of proteins, such as the soluble acyl-coenzime A binding protein (AcBP) in *Dictiostelium*^66^ and with starvation-induced secretion of AcBP in *Saccharomyces cerevisiae* and *Pichia pastoris*^67^,^68^. Furthermore, GRASP is involved in the Golgi bypass of αPS1 integrin during the stage 10B of *Drosophila* embryogenesis^69^. The GRASP mediated unconventional secretion pathway can be used to rescue the cell surface expression of the mutant ΔF508 in the cystic fibrosis transmembrane conductance regulator (CFTR)^15^, the most common mutation related with the cystic fibrosis disease, and this rescue only happens with the correct interaction between both proteins. GRASP is apparently also required for exporting of molecules of non-protein nature. In *C. neoformans,* deletion of the CnGRASP resulted in inefficient secretion of GXM, reduced capsule formation and attenuated virulence^17^. These data indicate that GRASP is also implicated in polysaccharide secretion via unconventional pathways and, consequently, fungal virulence. Properties including structural flexibility and related promiscuity can be further used to gain insight of how this class of protein can be so dynamically functional, with particular emphases in unconventional protein secretion.

The unexpected behavior of full-length CnGRASP in solution, indicating the presence of intrinsically disordered regions (IDRs) within its structure, might be essential in understanding the plethora of functions in which the protein is involved. The results here presented suggest that intrinsic disorder must then be kept in mind when one is dealing with processes related to GRASP function.

## Methods

### Bioinformatics

PHYRE2^70^ and I-TASSER^71^ were used for CnGRASP structure prediction, posteriorly refined using FG-MD^72^. The prediction of disordered segments was performed using VSL2B^73^, VL3 and VLXT^74^, and Ronn^75^ methods. SymPRED^76^, PredictProtein^77^, JPred^78^ and SSPro^79^ were used for the secondary structure prediction. The protein charge under physiological pH was estimated using Protein Calculator v3.4 (http://protcalc.sourceforge.net/).

### Protein expression and purification

*E. coli* Rosetta (DE3) cells harboring the pETSUMO-CnGRASP vector were grown at 310 K in LB medium containing kanamycin (50 μg/mL) and chloramphenicol (34 μg/mL), and expression was induced by isopropyl-β-D-thiogalactopyranoside (0.5 mM) for 16 hours at 291 K. The cells were harvested by centrifugation and the pellets stored at 251 K. Cells were resuspended in 25 mM HEPES, 150 mM NaCl, 1 mM CHAPS, 10% V/V Glycerol, pH 7.4 (buffer A) and lysed by sonication. The insoluble material was removed by centrifugation (14,000 × g for 25 minutes, in a temperature of 8 °C). CnGRASP was loaded onto a Ni-NTA superflow column (QIAGEN). The column was washed and the 6His-SUMO tag was removed using 2 mg of purified recombinant ULP-1 protease (for 3 hours, 283 K). CnGRASP (without 6His-SUMO tag) was collected in the flow-through fraction, concentrated by centrifugation in a Sorvall RC 6 plus centrifuge (Thermo Scientific) and the remaining contaminants were removed by size exclusion chromatography onto a Superdex 200 10/300 GL gel filtration column (GE Healthcare Life Sciences). For protein concentration, an Amicon Ultra-15 Centrifugal Filter with a NMWL of 10 kDa (Merck Millipore) was used.

### Circular Dichroism (CD)

Far-UV (190–250 nm) CD experiments were carried out in a Jasco J-815 CD Spectrometer (JASCO Corporation, Japan) equipped with a Peltier temperature control and using a quartz cell with a path length of 1 mm s. The experimental parameters were: scanning speed of 50 nm·min^−1^, spectral bandwidth of 1 nm, response time of 0.5 s and with separate measurements prior the average in order to avoid instability contributions in the final spectra. CnGRASP was dialyzed in 10 mM of Sodium Phosphate buffer, 10 mM NaCl, pH 8.0 and a final concentration of 7 μM was used. The software CDSSTR^30^,^31^ with an appropriate database available at DICHROWEB (http://dichroweb.cryst.bbk.ac.uk/html/home.shtml)^32^ was used for spectral deconvolution. Chemical stability experiments were performed in 25 mM HEPES, 10 mM NaCl, 1 mM CHAPS, 1mM DTT, 10% V/V Glycerol, pH 7.4 and increasing urea concentration. All the experiments were temperature controlled, fixed in 293 K. The synchrotron radiation circular dichroism (SRCD) spectra of CnGRASP (19 μM) were obtained at the UV-CD12 beamline located at the ANKA Synchtrotron (Germany). Three scans were collected over the wavelength range from 280 to 180 nm with 0.5 nm step size, 1.5 s dwell time in a 10 μm pathlength demountable Suprasil quartz cell (Hellma Ltd, UK); the baseline for each sample consisted of the buffer and all components present in the sample other than the protein. The effect of temperature was assessed by measuring SRCD spectra over the temperature range 20 to 85 °C, at 5 °C intervals, with an equilibration time of 5 min at each step. The spectra were processed using CDTools software^80^.

### Steady-State Fluorescence Spectroscopy

Fluorescence was monitored using a Hitachi F-7000 spectrofluorimeter equipped with a 150 W xenon arc lamp. The excitation and emission monochromators were set at 2.5 nm slit width in all experiments. The protein concentration was 10 μM. The chemical unfolding was performed using a fixed concentration of 1-anilino-8-naphthalenesulfonic acid (ANS–250 μM) in order to probe the exposure of hydrophobic regions of the protein upon urea addition. The excitation wavelength was set at 360 nm and the emission spectrum was monitored from 400 up to 650 nm. For tryptophan fluorescence experiments, the selective tryptophan excitation wavelength was set at 295 nm and the emission spectrum was monitored from 310 up to 400 nm. Fluorescence quenching using the water-soluble acrylamide as a quencher was done at 293 K and analyzed using the Stern-Volmer model^51^.

### Size-exclusion Chromatography

Size-exclusion chromatography was carried out using a Superdex 200 (HR 10/30) coupled to an *Äkta purifier* system (GE Healthcare). Apparent molecular mass and hydrodynamic radius (R_h_) were obtained from plots of K_av_ vs. log (R_h_) or log (MM) (molecular mass), where K_av_ is the partition coefficient. Standard proteins used were from the gel filtration calibration kit LMW and HMW (GE Healthcare) with the exception of human S100A12 that was purified as reported in the literature^81^. In order to estimate the correct molecular dimension, for these experiments, CHAPS were dropped off in the protein preparation.

### Limited proteolysis

Proteolysis sensitivity was assessed using bovine Trypsin (Sigma) in a molecular ratio (protein/trypsin) of 20/1 and 100/1. Conalbumin and Ovoalbumin (GE Life Science) were used as models of well-structured proteins for direct comparison. All reactions were checked by comassie stained 15% SDS-PAGE.

### Small Angle X-Ray Scattering (SAXS)

SAXS data were measured at the small-angle X-Ray scattering beamline of the National Synchrotron Light Laboratory (Campinas, Brazil)^82^. Samples were measured at the wavelength of 1.488 Å with detector/sample distance of 1,500 mm. Concentrations of purified CnGRASP of 0.5 mg/mL, 1 mg/mL and 2 mg/mL were used. The protein solutions and buffer were exposed in time-varying frames to monitor radiation damage and beam stability. Possible artifacts from buffer scattering were subtracted after the data being normalized to the intensity of the incident beam and corrected for detector response. The data analysis was carried out using ATSAS package^83^, more specifically the softwares GNOM^84^ and PRIMUS^85^.

### Differential Scanning calorimetry (DSC)

DSC experiments were done in a Nano-DSC II from Calorimetry Sciences Corporation, CSC (Lindon, Utah, USA). Samples were degassed under vacuum for 10 min before use and the data subtracted from buffer contributions. Scans were recorded from 10–95 °C at an average heating rate of 20 °C/hour, under pressure of 3 atm. Protein concentrations of 25, 50 and 100 μM were used. For comparison to a well-structured protein, a solution of 50 μM of Conalbumin (GE Life Science) was used as a control.

### Ultracentrifugation

A sucrose gradient ranging from 10 to 35% in a 25 mM Hepes/NaOH, 150 mM NaCl, pH 7.4 was prepared. The sucrose percentage was estimated by the difference in the diffraction indices. Samples of purified CnGRASP (less than 1 mg/mL) were loaded onto the gradient and centrifuged using a vertical rotor (Hitachi P65VT3) in a Hitachi 55P-72 ultracentrifuge (100,000 *g* for 4 h at 4 °C). Thyroglobulin, Conalbumin, BSA (GE Life Science) and Carbonic Anhydrase (Sigma Aldrich) were used as standard markers. The protein identification was carried out using absorbance at 280 nm and SDS-PAGE.

### Dynamic light scattering (DLS)

The CnGRASP DLS experiments were performed using a Nano-ZS dynamic light scattering system (Malvern Instruments Ltd, Malvern, UK). This system employs a 633 nm laser and a fixed scattering angle (173°). The experiments were carried out at 20 °C in a 25 mM Hepes/NaOH, 150 mM NaCL, 1 mM β-Mercaptethanol, 10% glycerol, pH 7.4 buffer. Before any measurement, the sample was centrifuged at 11,000 × g for 5 minutes at room temperature, and subsequently loaded into a quartz cuvette prior to measurement.

### Nuclear magnetic resonance (NMR)

^15^N-CnGRASP was expressed in minimum medium supplemented with ^15^N labeled ammonium chloride (Sigma Aldrich) and purified using the same procedure for the unlabeled sample. The protein was dissolved in 10% deuterium oxide in phosphate buffer pH 7.4 at a final concentration of 180 μM for NMR measurements. Glycerol (5% V/V) and beta-mercaptoethanol (5 mM) were present in the sample. NMR experiments were conducted in an AVANCE III HD Bruker spectrometer (Germany) operating at 600 MHz for ^1^H equipped with a triple resonance cryoprobe. The regular 1D spectrum was first obtained using the water suppression by excitation sculpting pulse sequence with gradients. A spectral width of 16 ppm and acquisition time of 3.4 s were set. A recycle delay of 2 seconds and delay for gradient recovery of 200 μs were used and 256 scans were recorded. For ^1^H -^15^N Heteronuclear Single Quantum Coherence (HSQC), the spectra were collected in increasing urea concentration (0, 2 and 4 M). For each experiment, 256 complex increments of 2048 complex data points were collected using 16 scans. The spectral widths were set to 14 ppm (^1^H) and 32 ppm (^15^N) and a relaxation delay of 2 seconds and delay for gradient recovery of 200 μs were used between scans.

## Additional Information

**How to cite this article**: Mendes, L. F. S. *et al*. New structural insights into Golgi Reassembly and Stacking Protein (GRASP) in solution. *Sci. Rep.* **6**, 29976; doi: 10.1038/srep29976 (2016).

## Supplementary Material

Supplementary Information
