# Supplementary material for: New structural insights into Golgi Reassembly and Stacking Protein (GRASP) in solution
Source: Sci Rep. 2016 Jul 20;6:29976. doi: 10.1038/srep29976 (PMC4951691; doi:10.1038/srep29976)
Supplement: Supplementary Information [file srep29976-s1.pdf]

Supporting Information

New structural insights into Golgi Reassembly and Stacking Protein (GRASP) in solution

Luís F. S. Mendes[1], Assuero F. Garcia[1], Patricia S. Kumagai[2], Fabio R. Morais,[3] Fernando A. Melo,[3] Livia K. Rosa e Silva[4], Marilene H. Vainstein[4], Marcio L. Rodrigues[5,6], Antonio J. Costa-Filho[1*]

1- Laboratório de Biofísica Molecular, Departamento de Física, Faculdade de Filosofia Ciências e Letras de Ribeirão Preto, Universidade de São Paulo, Ribeirão Preto, SP, Brazil
2- Departamento de Física e Informática, Instituto de Física de São Carlos, Universidade de São Paulo, São Carlos, SP, Brazil
3- Departamento de Física, Centro Multiusuário de Inovação Biomolecular, Instituto de Biociências, Letras e Ciências Exatas, Universidade Estadual Paulista Júlio Mesquita, São José do Rio Preto, Brazil
4- Centro de Biotecnologia, Federal University of Rio Grande do Sul, Porto Alegre, Brazil
5- Fundação Oswaldo Cruz - Fiocruz, Centro de Desenvolvimento Tecnológico em Saúde (CDTS), Rio de Janeiro, Brazil
6- Instituto de Microbiologia Paulo de Góes, Universidade Federal do Rio de Janeiro, Rio de Janeiro, Brazil

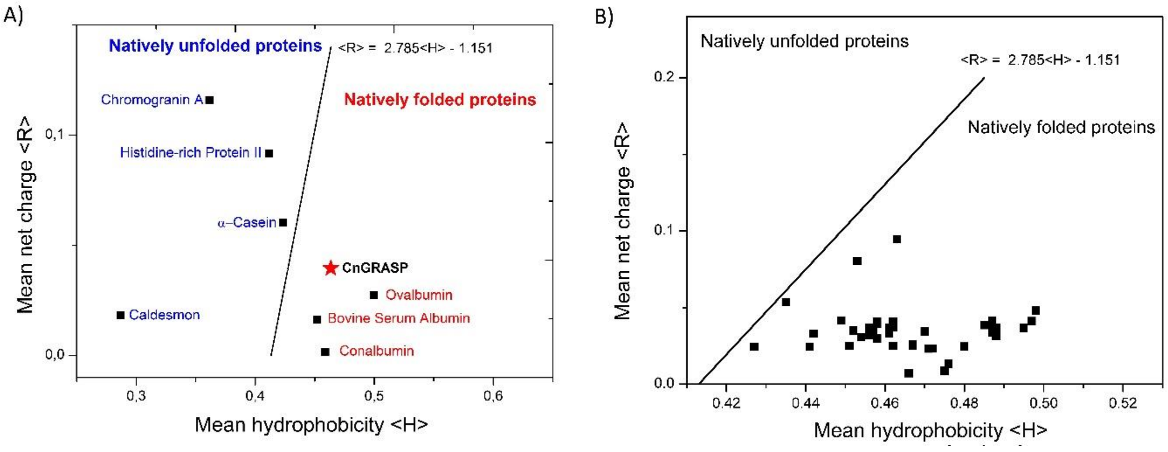

Fig S1. Uversky plot

A) Values of mean net charge (<R>) versus mean hydrophobicity (<H>) (taken from reference 25 of the manuscript) and/or calculated based on primary sequence collected from NCBI database. The red star locates the position of CnGRASP in the plot. B) It is worthwhile to notice that all proteins tested are located in the natively folded proteins side. The list with the GRASPs sequences can be founded in the end of the supporting text.

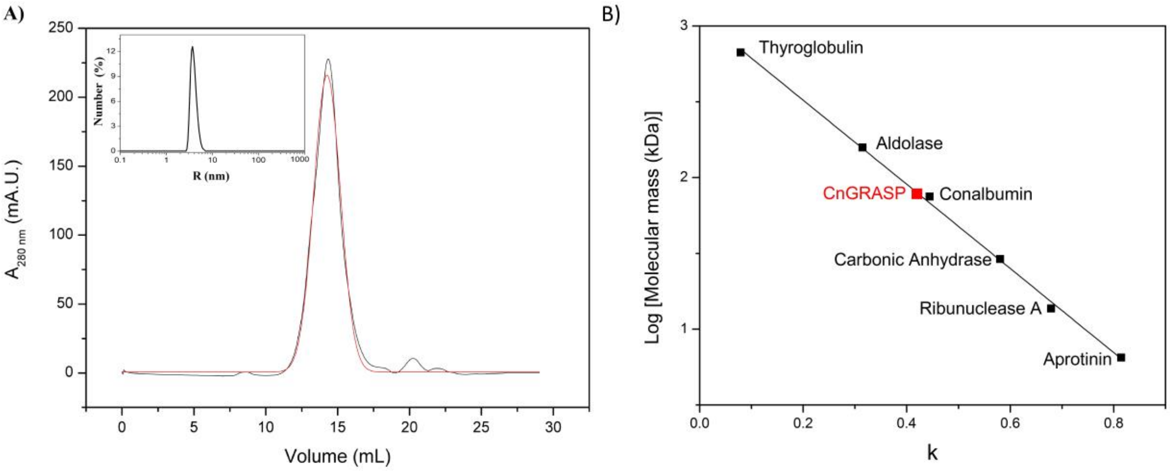

Fig S2. Determination of the apparent molecular mass of CnGRASP by size exclusion chromatography (SEC)

A) Elution pattern of purified CnGRASP and a Gaussian fit (red) which suggest a homogeny elution. In the inset on left, it is a DLS result showing that the sample is monodisperse and centered in a value close to the one observed in the SEC results. The choice for the SEC value is because we could not satisfactory evaluate a viscosity coefficient for R_h determination. (Using the water viscosity value, we still rescue an R_h that gives a molecular weight of 68.9 kDa in the Siegel and Monte model, consistent with a dimer conformation). B) Partition coefficient as function of the logarithm of molecular mass, fitted with a linear function where the adjusted R-square was 0.998.

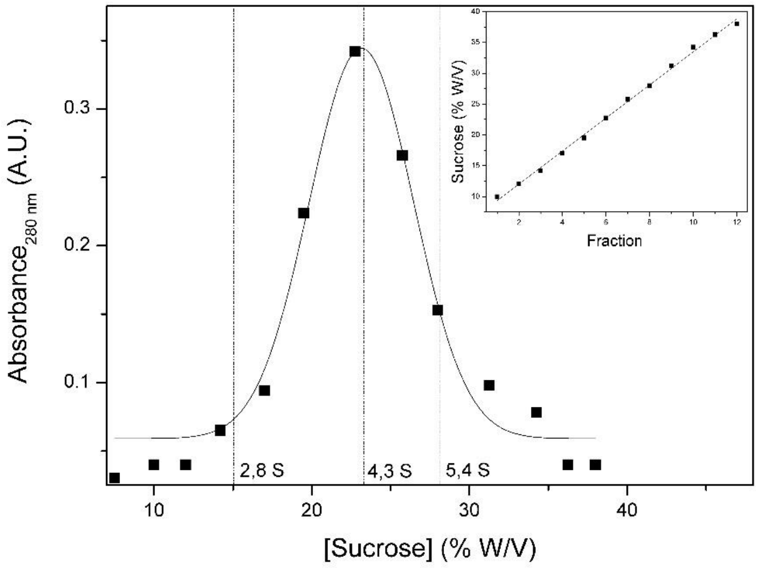

Fig S3. Elution profile of CnGRASP in a sucrose gradient after ultracentrifugation
The dotted lines indicate the elution of the standard proteins. The linearity of the sucrose gradient was monitored using the diffraction index variation and is located in the inset.

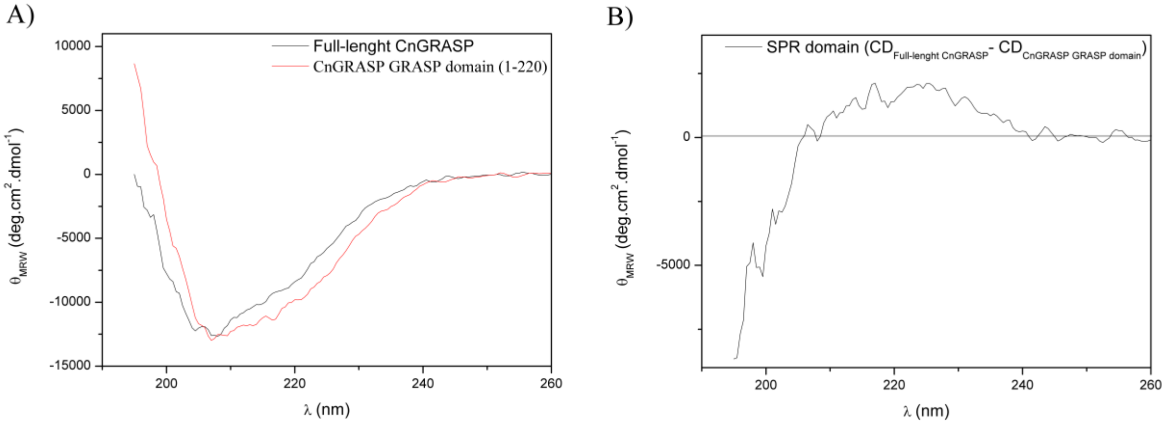

Fig S4. CD analyses of CnGRASP domains
A) CD signal of full-length CnGRASP and from our GRASP domain construction (1-220), prepared in the same way as the native one. The signals are normalized in θ_{MRW} units. B) The theoretical SPR signal obtained from the CD signals presented in A. We supposed that the GRASP domain and the SPR domain are independent structures, so they have independent CD signals that, together, gives the full-length CD one. This cannot be used as a definitive prove, but as indication, together with our other results, that the SPR domain has the full intrinsically disordered patter

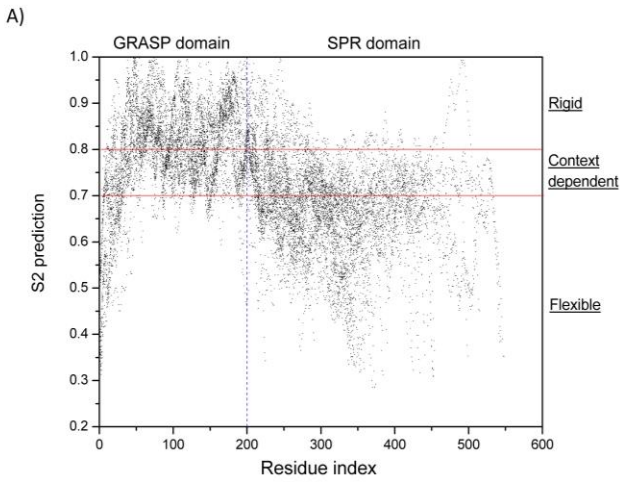

Fig S5. Disorder prediction along GRASP family

A) Backbone flexibility prediction at the residue-level in the form of backbone N-H S^2 order parameter values using DynaMine. Zone propensity corresponding to a rigid, flexible and context dependent limit states are detached. B) Disorder prediction using VSL2 predictor. It is worthwhile to notice that the SPR domain has a high disordered/flexibility propensity along all GRASPs tested, a tendency not observed for the GRASP domain.

Table S1. Deconvolution results of CnGRASP CD data using DICHROWEB

Program	Data base	α-helix 1	α-helix 2	β - sheet 1	β – sheet 2	Turn	Disordered	NRMSD
CDSSTR	SET 3	0.11	0.11	0.14	0.1	0.22	0.32	0.021
CDSSTR	SET 6	0.10	0.09	0.11	0.08	0.18	0.44	0.027
CDSSTR	SP175	0.09	0.12	0.14	0.1	0.14	0.40	0.01
**Mean value**		10%	10.7%	13%	9.3%	18%	38.7%	

Table S2: Secondary structure prediction content in CnGRASP using SymPRED, Jpred and SSpro

Predictors	α-helix	β-sheet	Loop
PROFsec	8%	25%	67%
Jpred	10%	28%	62%
SSpro	11%	24%	65%
**Mean value**	**9.7%**	**25.7%**	**65.7%**

List of GRASP sequences used in this work (figure S1 and S5), collected from the NCBI database and in a FAST format.

>gi|657135458|gb|KEG07653.1| Golgi reassembly stacking protein, partial [Trypanosoma grayi]

>gi|151942478|gb|EDN60834.1| grasp65 (Golgi reassembly stacking protein of 65kd)-like protein [Saccharomyces cerevisiae YJM789]

>gi|52345514|ref|NP_001004805.1| golgi reassembly stacking protein 1, 65kDa [Xenopus (Silurana) tropicalis]

>gi|52345500|ref|NP_001004798.1| golgi reassembly stacking protein 2, 55kDa [Xenopus (Silurana) tropicalis]

>gi|148233199|ref|NP_001080519.1| golgi reassembly stacking protein 2, 55kDa [Xenopus laevis]

>gi|70833735|gb|EAN79237.1| Golgi reassembly stacking protein (GRASP homologue), putative [Trypanosoma brucei brucei strain 927/4 GUTat10.1]

>gi|666434722|gb|KEY82217.1| golgi family reassembly stacking protein [Aspergillus fumigatus var. RP-2014]

>gi|635510258|gb|KDE82201.1| golgi family reassembly stacking protein [Aspergillus oryzae 100-8]

>gi|119631632|gb|EAX11227.1| golgi reassembly stacking protein 2, 55kDa, isoform CRA_c [Homo sapiens]

>gi|629675408|ref|XP_007799201.1| putative golgi reassembly stacking protein [Eutypa lata UCREL1]

>gi|557727781|dbj|GAD93621.1| Golgi reassembly stacking protein, putative [Byssochlamys spectabilis No. 5]

>gi|573987781|ref|XP_006671811.1| golgi reassembly stacking protein [Cordyceps militaris CM01]

>gi|562972405|gb|ESW98139.1| Golgi reassembly stacking protein [Ogataea parapolymorpha DL-1]

>gi|512188996|gb|EPE04765.1| golgi family reassembly stacking protein [Ophiostoma piceae UAMH 11346]

>gi|477526341|gb|ENH78208.1| golgi reassembly stacking protein [Colletotrichum orbiculare MAFF 240422]

>gi|471559242|gb|EMR61688.1| putative golgi reassembly stacking protein [Eutypa lata UCREL1]

>gi|425769460|gb|EKV07952.1| Golgi reassembly stacking protein, putative [Penicillium digitatum PHI26]

>gi|358367117|dbj|GAA83736.1| golgi reassembly stacking protein [Aspergillus kawachii IFO 4308]

>gi|396483182|ref|XP_003841646.1| similar to golgi reassembly stacking protein [Leptosphaeria maculans JN3]

>gi|346320588|gb|EGX90188.1| golgi reassembly stacking protein [Cordyceps militaris CM01]

>gi|327348935|gb|EGE77792.1| golgi reassembly stacking protein [Ajellomyces dermatitidis ATCC 18188]

>gi|326469524|gb|EGD93533.1| golgi reassembly stacking protein [Trichophyton tonsurans CBS 112818]

>gi|326461673|gb|EGD87126.1| golgi reassembly stacking protein [Trichophyton rubrum CBS 118892]

>gi|320593115|gb|EFX05524.1| golgi reassembly stacking protein [Grosmannia clavigera kw1407]

>gi|325093153|gb|EGC46463.1| golgi reassembly stacking protein [Ajellomyces capsulatus H88]

>gi|320040036|gb|EFW21970.1| golgi reassembly stacking protein [Coccidioides posadasii str. Silveira]

>gi|149022191|gb|EDL79085.1| golgi reassembly stacking protein 2 [Rattus norvegicus]

>gi|83638659|gb|AAI09692.1| Golgi reassembly stacking protein 2, 55kDa [Bos taurus]

>gi|212529034|ref|XP_002144674.1| Golgi reassembly stacking protein, putative [Talaromyces marneffei ATCC 18224]

>gi|119584963|gb|EAW64559.1| golgi reassembly stacking protein 1, 65kDa, isoform CRA_c [Homo sapiens]

>gi|51259254|gb|AAH78731.1| Golgi reassembly stacking protein 2 [Rattus norvegicus]

>gi|157117495|ref|XP_001658795.1| golgi reassembly stacking protein 2 (grasp2) [Aedes aegypti]

>gi|557866393|gb|ESS69592.1| Golgi reassembly stacking protein [Trypanosoma cruzi Dm28c]

>gi|472581853|gb|EMS19568.1| golgi reassembly stacking protein 2 [Rhodosporidium toruloides NP11]

>gi|470242986|ref|XP_004355215.1| golgi reassembly stacking protein [Dictyostelium fasciculatum]

>gi|401428022|ref|XP_003878494.1| putative Golgi reassembly stacking protein (GRASP homologue) [Leishmania mexicana MHOM/GT/2001/U1103]

>gi|349804463|gb|AEQ17704.1| putative golgi reassembly stacking protein subunit, partial [Hymenochirus curtipes]

>gi|398021825|ref|XP_003864075.1| Golgi reassembly stacking protein (GRASP homologue), putative [Leishmania donovani]

>gi|389610007|dbj|BAM18615.1| golgi reassembly stacking protein 2 [Papilio xuthus]

>gi|154344180|ref|XP_001568034.1| putative Golgi reassembly stacking protein (GRASP homologue) [Leishmania braziliensis MHOM/BR/75/M2904]

>gi|353233065|emb|CCD80420.1| putative golgi reassembly stacking protein 2 (grasp2) [Schistosoma mansoni]

>gi|387849038|ref|NP_001248636.1| Golgi reassembly-stacking protein 2 [Macaca mulatta]

>gi|60302830|ref|NP_001012612.1| Golgi reassembly-stacking protein 2 [Gallus gallus]

>gi|41053495|ref|NP_956997.1| Golgi reassembly-stacking protein 2 [Danio rerio]

>gi|197097294|ref|NP_001126857.1| Golgi reassembly-stacking protein 2 [Pongo abelii]

>gi|213515012|ref|NP_001133999.1| Golgi reassembly-stacking protein 2 [Salmo salar]
